# Retrospective cross-sectional study of 34 cases of pernicious anemia at Mohammed V Military Training Hospital, Morocco

**DOI:** 10.11604/pamj.2023.45.79.34204

**Published:** 2023-06-08

**Authors:** Youssra Azzouz, Soukaina Abidi, Saliha Chbicheb

**Affiliations:** 1 Oral Surgery, International Faculty of Dentistry, International University of Rabat, Rabat, Morocco,; 2 Oral Surgery, Faculty of Dentistry, Mohammed V University, Rabat, Morocco

**Keywords:** Anemia, pernicious, vitamin B12, anti-intrinsic factor antibody, anti-parietal cell antibody

## Abstract

**Introduction:**

pernicious anemia is an autoimmune disease characterized by atrophic gastritis due to malabsorption of vitamin B12. Certain oral manifestations, such as Hunter´s glossitis and burning mouth syndrome, may precede the onset of this anemia. The aim of this study is to describe the clinical presentation, para-clinical aspects, the treatment, and the evolution of the pernicious anemia (PA) after treatment.

**Methods:**

retrospective study conducted at the Department of Haematology and Internal Medicine B of the Mohammed V Military Training Hospital in Rabat between January 2009 and December 2018. Thirty-four patients were enrolled with vitamin B12 deficiency, non-regenerative macrocytic anemia, a positive anti-intrinsic factor antibody and anti-parietal cell antibody and a histological diagnosis of atrophic gastritis in the presence or not of Helicobacter pylori. The qualitative variables were expressed in numbers and percentages, and the quantitative variables in mean and standard deviation. Multivariate analysis used the Fischer test; it was considered significant for a p < 0.05 value.

**Results:**

thirty-four cases were studied; the population study consists of 56% (n=19) of men and 44% (n=15) of women. The average age was 54.88± 9.14. The clinical manifestations of pernicious anemia are dominated by megaloblastic anemia 85.3% (n=29), followed by digestive 58.8%(n=20) and oral 55.9% (n=19) signs. Neurological manifestations were rarely found in 41% (n=14). Hunter´s glossitis 37% (n=7), stomatodynia 11% (n=2) were the most common oral manifestations accompanying pernicious anemia. The evolution was favorable in 79.4% (n=27) patients under substitution therapy with vitamin B12.

**Conclusion:**

dentists´ involvement in the diagnosis of pernicious anemia is based on changes in oral mucous membranes, which have been reported in 55.9% of all patients. These oral changes may occur in the absence of symptomatic anemia.

## Introduction

Pernicious anaemia (PA), also known as Biermer´s disease [1], is a macrocytic anaemia caused by a vitamin B12 (cobalamin) deficiency, which, in turn, is the result of intrinsic factor (IF) deficiency, a protein that binds cobalamin and thereby enables its absorption at the terminal ileum [2]. The deficiency of IF is a consequence of the presence of atrophic gastritis, which results in the destruction of the oxyntic mucosa and thus the loss of parietal cells, which normally produce hydrochloric acid as well as IF [3]. Despite the advances in understanding the pathogenesis and molecular biology, diagnosis of PA is still challenging because of its diverse clinical presentations, and the limitations of the available diagnostic tools for the evaluation of cobalamin status and the presence of chronic autoimmune atrophic gastritis. When the disease remains undiagnosed and untreated for an extended period, it may lead to neurological complications and even fatal anemia. In Africa, the pathology is increasingly described [4]. The aim of this study is to describe the clinical presentation, para-clinical aspects, the treatment and the evolution of the pernicious anemia (PA) after treatment.

## Methods

**Setting and study design:** it is a retrospective cross-sectional study, including 34 observations of pernicious anemia managed in the Department of Internal Medicine B and the Clinical Haematology Department of the Mohammed V Military Training Hospital of Rabat between 2009 and 2018.

**Data sources:** these observations were selected from the hospital discharge summary of all patients hospitalized during the period considered.

**Participants:** only patients with B12 deficiency and a proven pernicious anemia were retained after file review by two independent “Seniors” internists.

**Study size:** thirty-four patients with proven B12 deficiency related pernicious anemia were selected.

**Inclusion and exclusion criteria:** the patients included in this study all had pernicious anemia identified by at least three of the four major diagnostic criteria: low serum vitamin B12 levels, megaloblastic anaemia, gastric atrophy, and the presence of antibodies to gastric parietal cell or IF. They were excluded from this study the other causes of vitamin B12 deficiency, of anemia and the other types of gastritis. Various data were collected and analyzed for each patient: epidemiological data (age, sex, geographic origin), background, clinical examination (including study of different syndromes: anemic, digestive, stomatological and neurological), the blood count, serum values of vitamin B12 and folic acid, autoantibodies gastric parietal cells, and anti-intrinsic factor, the myelogram, gastroscopy data with biopsies (including the presence or absence of *Helicobacter pylori*), treatment modalities and the elements of the monitoring of each patient. Serum B12 concentrations (normal: 200-1100 pg/ml) and folate (normal: 3.5-8 ng/ml) were determined using the kit: Radio Immuno Assay, Bayer Corp. (New York, USA), anti-FI and anti-CPG antibodies have been tested by kit: Elisa, Bayer Corp. (New York, USA); and Haematological parameters using an electronic counter: Technikon H1, Bayer Corp. (NewYork, USA).

**Quantitative variables, and statistical methods:** the qualitative variables were expressed in terms of numbers and percentage and the quantitative variables in mean and standard deviation. The analysis of the results was carried out with the SPSS 21.0 (statistic package for social science) software for Windows 10. The graphs were made with the Microsoft Office Excel 2016 software. Multivariate analysis used the Fischer test; it was considered significant for a p < 0.05 value.

## Results

**Figure 1:**
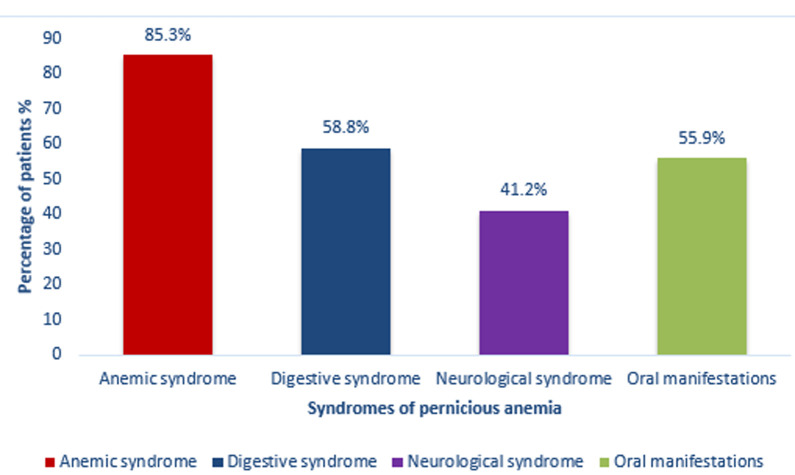
distribution of different syndromes of pernicious anemia

The population study consists of 56% (n=19) men and 44% (n=15) women, the median age at diagnosis was 54.88 ± 9.14. In terms of geographical origin, the patients were all Moroccan, distributed over various regions, with a regional predominance Rabat-Salé-Kenitra. The different clinical syndromes identified were anemic, digestive, neurological syndrome and oral manifestations (Figure 1). The anemic syndrome was the most common symptom 85% (n=29) with the following events: gradual installation 10.3% (n=5), pallor 3.4% (n=1), tachycardia 4% (n=2), gradual installation with pallor 27.6% (n=9), jaundice 10.3% (n=5), gradual installation with pallor and asthenia 20.7% (n=7). Digestive syndrome was present in 58.8% (n=20) of patients with clinical manifestations including anorexia 40% (n=10), dyspeptic disorders 35% (n=9) and emesis 5% (n=1). Neurological syndrome was present in 41.2% (n=14) patients with clinical manifestations mainly represented by paresthesia 35.7% (n=12), sensitivity disturbances 21.4% (n=7) and vertigo 14.3% (n=4). Psychiatric disorders were not observed in any of our patients. Oral manifestations were present in 55.9% (n=19) patients (Table 1).

**Table 1:** oral manifestations of pernicious anemia (n = 19)

Symptoms and signs	No. (%) of patients
Hunter's glossitis	37%(n=7)
Burning sensation	11% (n=2)
Hunter's glossitis + burning sensation	26% (n=5)
Xerostomia	11% (n=2)
Papillary atrophy	5% (n=1)
Mouth sores	5% (n=1)
Hunter’s glossitis+ recurrent aphthous stomatitis	5% (n=1)

In complementary examinations, anemia was noted in 85.3% (n=29) of our patients, haemoglobin level was <5g/dl in 26% (n=9) patients, between 5 and 10 in 56% (n=19) patients and >10 in 18% (n=6) patients. Anemia was nonregenerative in 91.2% (n=31) patients, macrocytic in 97% (n=33) of cases, while in 3% (n=1) of cases it was normocytic. Thrombocytopenia was observed in 3% (n=1) patient. Leukopenia was observed in 9% (n=3) patients. Leukopenia and thrombocytopenia were noted in 17% (n=6) patients. The myelogram was performed in 16 patients, the marrow was rich in 47.1% (n=16) cases, a medullary megaloblastosis was noted in 40% (n=4) patients, cellular gigantism in erythropoiesis in 20% (n=2) patients and an association of the two in 40% (n=4) patients. Level of vitamin B12 was low in all of our patients, 100% (n=34). Normal dose of folic in 90% (n=19) of patients and high dose in 10% (n=2) of patients (Table 2). Autoantibody detection, anti-intrinsic factor and anti-parietal cells were performed in 85.3% (n=31) patients. Esophagogastro duodenoscopy (EGD) was performed in 64.7% (n=22) of patients. Gastritis was found in 36% (n=8) of patients. Gastritis with positive *Helicobacter pylori* was found in 32% (n=7) of patients. Gastritis with negative *Helicobacter pylori* was found in 32% (n=7) of patients. The homocysteine assay was not performed in our patients (Table 3). All our patients were treated with vitamin B12 injections at 5000 μg per day until the reticulocyte crisis, then 5000 μg per week until normalization of hemoglobin level, then 5000 μg per month for life. A treatment with iron supplements was associated in 26% (n=9) cases. In all patients, normalization of serum B12 concentrations and correction of blood count abnormalities were observed. In 79.4% (n=27) patients who returned for control, gastric tumours were not detected despite iterative gastroscopies, with an average follow-up of 1.5 ± 2 years.

**Table 2:** laboratory findings at the time of diagnosis

Findings	No. of patients (%)
Hemoglobin (g/dL)	
<5g/dl	26% (n=9)
5-10g/dl	56% (n=19)
>10g /dl	18% (n=6)
Mean corpuscular volume (MCV) (fL)	
<100	3% (n=1)
>100	97% (n=33)
Leukopenia (< 4.0 × 10^9^ /L)	9% (n=3)
Platelets (× 10^9^/L) < 50	3% (n=1)
Leukopenia + thrombocytopenia	17% (n=6)
Myelogram	47.1% (n=16)
Medullary megaloblastosis	40% (n=4)
cellular gigantism	20% (n=2)
Medullary megaloblastosis+ cellular gigantism	40% (n=4)
Vitamin B12 (pg/mL)	
50-100	100% (n=34)
Plasma folate level (ng/mL)	
2-20 ng/mL	90% (n=19)
>20ng/ml	10% (n=2)

**Table 3:** findings of serum antibodies and *H. pylori* testing

Findings	No. of patients (%)
Antibodies	87.3% (n=31)
Anti-intrinsic factor antibody (+)	23.5% (n=8)
Anti-parietal cell antibody (+)	20.5% (n=7)
Both antibodies (+)	26.4% (n=9)
Both antibodies (-)	20.5% (n=7)
*H. pylori* testing	64.7% (n=22)
Positive	32% (n=7)
Negative	32% (n=7)

## Discussion

The aim of our study is to provide description of the clinical presentation, para-clinical aspects, the treatment, and the evolution of the pernicious anemia after treatment in 34 cases. Overall, the clinical manifestations of pernicious anemia in our series are dominated by megaloblastic anemia, followed by digestive and oral signs. Neurological manifestations were not frequent. All patients present a low blood levels of vitamin B12. The normalization of serum B12 concentrations was observed in patients under substitution therapy with vitamin B12. Epidemiologically, the average age of our patients was 54.88 years, as other studies produced in developing countries which also showed 58.5 years [5] and 51 years [6]. A male predominance was found in our cases, similar to a previous report by Maazouna *et al*. [7] and Maamar *et al*. [8]. This can be explained by the fact that our study was carried out in a military hospital, where the recruitment is predominantly male. However, a female predominance has been reported by Ndiaye *et al*. [6]. The revealing events were dominated by anemic syndrome associated to lingual inflammation, digestive and neurological disorders in our study as for Koulidiati *et al*. Ndiaye *et al*. or Maamar *et al*. [5,6,8]. Oral signs have been reported in 55.9% of our patients. These oral changes may occur in the absence of symptomatic anemia or macrocytosis as they may precede many systemic indicators of B12 deficiency. In the series of Diop *et al*. [4] the mode of revelation in 2 patients was a glossitis and macrocytosis without anemia. Paresthesia was mostly found among our patient, and it is so described as an important physical sign throughout the literature [8]. The high prevalence of anemia in the African series [6,9] can be explained by the delay between first clinical presentation and physician consultation. This fact may also be related to long-term clinical resistance associated with the lack of technical facilities in developing countries [9].

Conventionally, pernicious anemia is manifested with macrocytosis, with a mean corpuscular volume >100 fl [6,10]. But there are situations that can mask the macrocytosis, which complicates the diagnostic process, like the case of iron deficiency, microcytic anemia [11] and anemia of inflammatory origin. A dosage of vitamin B12 was low in all of our patients 100% (n=34) which agrees with the Koulidiati and *et al*. study where they observed a Vitamin B12 Deficiency in all patients (100% of cases) [5]. Our study showed positivity of anti-intrinsic factor antibodies in the majority of cases. However, despite their high PA specificity, these antibodies may be absent in 30-50% of cases [12]. Histologically, the main aspect noted in our study was fundal atrophic gastritis in 95.4% of cases. This is an almost constant diagnostic criterion, reported with a high frequency in the majority of published studies [7,6,13].

The specific treatment based on the prescription of vitamin B12 for life, as indicated in the literature, was offered to all our patients. The parenteral route was the only one used in our series, all our patients were on injectable vitamin B12. Currently, the route of supplementation, with well-codified modalities, remains the injectable route (hydroxocobalamin in the form of ampoules dosed at 5000 μg [14]). However, the oral route is the most used in many years in Sweden and Canada [13-15]. The evolution after treatment was marked by a reticulocyte crisis with a normalization of all the parameters of the blood count obtained for all patients after a treatment of 03 months, which is in accord with the results obtained in the series of Ndiaye *et al*. [6], Maamar *et al*. [8]. In the absence of reticulocyte crisis and therefore of response, it is essential to look for another superadded cause of anemia, such as an associated iron deficiency. Our study has some limitations that should be mentioned. The first limitation is the lack of some data obtained from history, physical examination, paraclinical assessment and information on the long-term evolution of patients. The second limitation is the loss of follow-up for some patients, only 79.4% (n=27) of patients who continue to be assessed.

## Conclusion

The diagnosis of pernicious anemia remains challenging because of its diverse clinical manifestations and the limitations of currently available diagnostic tools. Oral lesions are among the most common initial symptoms, changes in oral mucous membranes have been reported in 55.9% of all patients with pernicious anemia. The dentist, who is often consulted first, has a prime opportunity and responsibility to contribute to diagnosis.

### What is known about this topic

- *Pernicious anaemia is an autoimmune disease of multifactorial aetiology involving environmental and immunological factors, It is the most common cause of cobalamin deficiency anaemia worldwide;*
- *The clinical presentation of pernicious anemia is often insidious and the classic presentation consists of a triad of jaundice, glossitis, and myeloneuropathy;*
- *Glossitis is a frequent sign of megaloblastic anaemia, with the patient displaying a painful, smooth, red tongue*.

### What this study adds

- *The clinical and biological manifestations of pernicious anemia are extremely varied, Most of our patients exhibited symptoms of anemia followed by digestive syndrome with anorexia and dyspeptic symptoms;*
- *According to our results, glossitis (Hunter glossitis) is the most common symptom; it may occur in the absence of symptomatic anemia or macrocytosis;*
- *Diagnosis of pernicious anemia was confirmed in our patients in the presence of chronic anemia with neurological manifestations, low blood levels of vitamin B12 and a positive anti-intrinsic factor antibodies test*.
